# Liver Trauma in the Kitchen: Preparing Whipped Cream with a Siphon Is Not without Risk

**DOI:** 10.1155/2015/213039

**Published:** 2015-06-07

**Authors:** Jeremy Bourenne, Béatrice Eon, Fouad Bouzana, Dominique Lambert, Estelle Jean, Pierre Michelet, Marc Gainnier

**Affiliations:** ^1^Reanimation des Urgences et Medicale, CHU la Timone 2 Marseille, Aix-Marseille Université, 13385 Marseille, France; ^2^Service d'Accueil des Urgences, CHU la Timone 2 Marseille, Aix-Marseille Université, 13385 Marseille, France

## Abstract

We report the case of a 36-year-old woman suffering from liver injury caused by the malfunction of a whipped cream siphon. When this patient handled the whipped cream siphon, the screwed metallic upper part of the siphon was suddenly dissociated from its base and came violently striking her right hypochondrium. At first, the severity of injury was underestimated. Subsequently, due to the persistence of pain experienced by the patient, an abdominal CT scan was performed. It highlighted a severe liver injury with rupture of a branch of the hepatic artery. The evolution was favorable after completion of an embolization and a secondary capsular rupture.

## 1. Introduction

Most causes of severe liver injury occur during car accidents, falls from great height, and all causes of high kinetic trauma. These injuries are often life-threatening because of the frequency of hemorrhagic shock. The clinical presentation is often very suggestive because abundant hemoperitoneum is frequently observed with ultrasound resulting in immediate surgery for hemostasis. If the patient is hemodynamically stable, an abdominal CT scan can be performed showing active bleeding. In such situations, the treatment is now based on interventional radiology with embolization of the bleeding vessel.

## 2. Case Presentation

**Figure 1:**
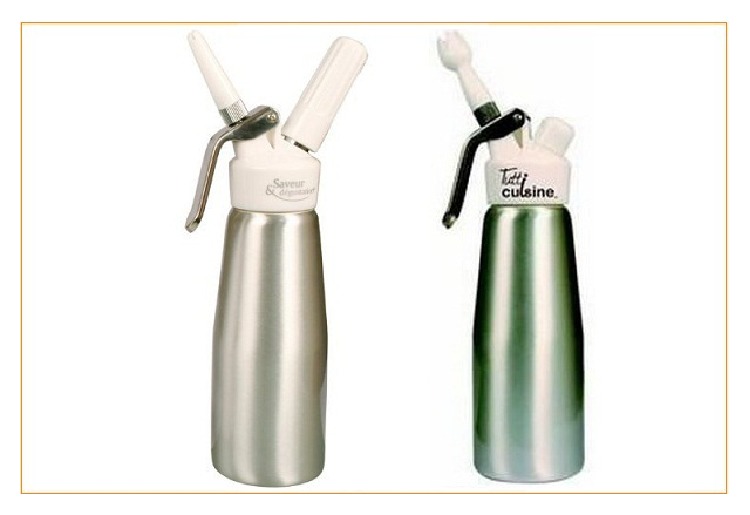
Whipped cream siphon implicated.

A 36-year-old woman is victim of abdominal blunt trauma caused by the top of a whipped cream siphon after she tried to screw this part while the gas cap was in place (Figure 1). The gas cap was inserted properly in the siphon, as in the user guide. During the screwing of the upper part of the siphon, the gas contained in the capsule was released, resulting in the projection of the upper part of the siphon on her right hypochondrium.

The patient subsequently experienced severe abdominal pain. Her husband immediately rushed his wife to the emergency department.

On admission and after clinical examination, the emergency physician prescribed symptomatic treatment of pain with acetaminophen. X-rays performed in emergency eliminated broken ribs. The chest radiograph was normal. The clinical picture presented by this patient was considered as a minor accident with no major consequences. No more investigations were performed. The patient was sent home.

In the night following the first emergency admission, the pain was becoming increasingly important.

The patient's husband was very worried. He brought his wife back to the emergency department.

**Figure 2:**
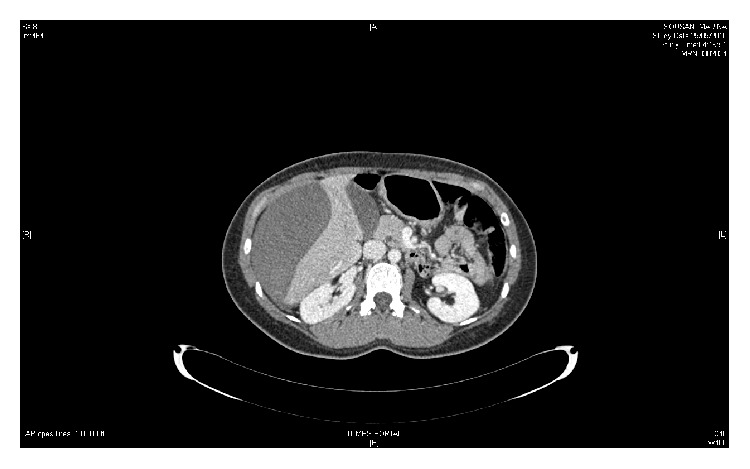
Abdominal CT scan with subcapsular hematoma and active bleeding.

**Figure 3:**
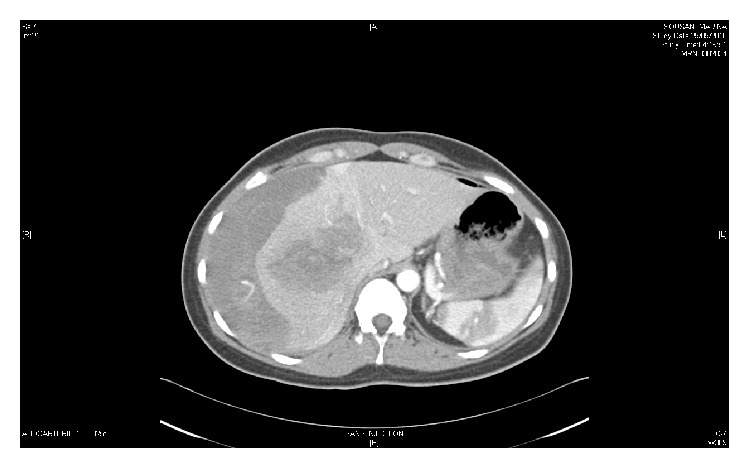
Subcapsular hematoma in abdominal CT scan without contraste.

At admission, he insistently claimed further investigations. At that time, the clinical examination by a novel emergency physician showed a large hematoma located on her right hypochondrium and on the anterior chest. Vital parameters were the following: heart rate 110 beats/min, blood pressure 90/46 mmHg, pulse oximetry (SpO2) 100%, and respiratory frequency 25 breaths/min. Patient's pain was treated with nefopam and tramadol. She received volume expansion from 2000 mL crystalloids, transfused of 3 red blood cells and 3 plasmas. A thoracoabdominal CT scan (Figures 2 and 3) was then performed in emergency, revealing a subcapsular hematoma of the liver associated with a contusion of the right liver in segment number 8. The radiologist also noted active bleeding at the right branch of the hepatic artery vascularising segment number 8. Treatment consisted of superselective embolization of the hepatic artery branch responsible for the bleeding. Hypotension and acute anemia required volume expansion and transfusion of 3 units of blood. No vasopressors were administered. The patient was admitted after embolization in the intensive care unit for monitoring. During 48 hours, hemodynamics and clinical status remained stable. At the 48th hour, the chest and abdominal pain recurred associated with tachycardia and dyspnea with no signs of acute anemia clinically and biologically (hemoglobin concentration stable). On clinical examination the hemodynamic is stable (blood pressure 130/60 mmHg, no mottling), and abdominal palpation found a defense in the right hypochondrium. A new abdominal CT scan was performed. It highlighted a hemoperitoneum due to rupture of the subcapsular hematoma into the peritoneal cavity without any new active bleeding. No surgical indication was retained by the surgeons consulted. The patient improved spontaneously in intensive care.

The fourth day of admission to intensive care, an abundant right pleural effusion was present on chest radiography. After the effusion was confirmed by pleuro-pulmonary ultrasound, the pleural drainage evacuate 1500 mL of transudate. On the fifth day, the patient presented again with dyspnea, chest pain, tachycardia, and agitation. Pulmonary embolism was suspected because no prophylaxis of thromboembolic disease has been prescribed for the first 3 days of admission for preventing the hemorrhagic risk.

She receives a mechanical prevention by intermittent compression of inferior members. A new chest CT scan with contrast was made and it did not show any evidence of pulmonary embolism but increased pleural effusion.

Because of the importance of liver injury and violence of the trauma, a right diaphragmatic rupture was suspected but Magnetic Resonance Imaging performed showed no lesions in favor of this hypothesis. The correct subsequent evolution did not indicate a videothoracoscopy for the thoracic surgeons consulted.

On the tenth day, she left the intensive care unit for surgery unit. There are no other complications later in hospitalization, liver function was normalized, and no transfusion was performed. She received only analgesic and iron supplementation. She left the surgery after 23 days of hospitalization. Two months after discharge from the hospital, the patient was well and complained of very light abdominal pain. Control CT scan showed parenchymal defects in segment 8, the site of embolization, associated with a minor perihepatic hematoma.

## 3. Discussion

We did not find any serious blunt liver injuries caused by a whipped cream siphon in the literature. Blunt traumas of the abdomen are often encountered in road accidents with high velocity traumatism. In these circumstances, the large and rapid increase in abdominal pressure caused by the impact of a blunt agent plays an important role in the risk of liver trauma. This has been described, for example, with airbags [1, 2]. In the case of this patient, the mechanism of the injury was explained in great detail by the husband at the first consultation at the emergency department. Clearly, during this first consultation, the mechanism of injury and the importance of pain experienced by the patient were not sufficiently taken into account, probably because of the absence of visible lesions (no wounds, no hematoma, and no penetration) that might raise suspicion of significant internal injury.

In violent abdominal trauma, multiple trauma, or in hemodynamically unstable patient, abdominal echography or abdominal CT is immediately realized by all clinicians to investigate intra-abdominal lesions [3, 4]. However, when the circumstances of the abdominal trauma are unusual or in hemodynamically stable patient the problem of the indication of imaging arises. One of the main objectives of the clinical evaluation in blunt abdominal trauma is to determine the velocity of trauma for indicating more imaging investigation.

Clinical examination of a trauma patient often underestimates intra-abdominal lesions in 20 to 40% of cases [5, 6]. A systematic Focused Assessment with Sonography for Trauma (FAST) found an incidence of 35% of liver lesion in blunt abdominal trauma hemodynamically stable patient [7]. A large prospective study found a sensibility of 43% and a specificity of 99% of FAST in hemodynamically stable abdominal trauma [8]. Another study performed a systematic whole body imaging in trauma patients without obvious sign of injury and found abnormalities in 7,1% of abdominal CT scans [9]. These data highlight the importance of early systematic imaging when the circumstances of abdominal blunt trauma are not well understood. This case report illustrates this problem.

During the second admission to the emergency ward, the hematomas on the chest and on the abdomen were then present. Therefore, the diagnosis of severe blunt thoracoabdominal trauma was made. After the diagnosis had been confirmed by CT scan, the management was based on a selective embolization of a branch of the hepatic artery. Liver lesions management is now fairly well standardized in hemodynamically stable or unstable patient. When the patient is stable, a simple monitoring is sufficient but when a full active leak of contrast material is objective, endovascular treatment is required when possible. This approach is now particularly recommended in the case of liver trauma hemodynamically stable [10, 11]. The evolution is generally favorable. The other originality of this case report is the secondary rupture of the capsular hematoma in the peritoneal cavity.

## 4. Conclusion

This case emphasizes the possibility of severe liver injury induced by a high velocity object projected on the abdomen next to the liver with early clinical manifestations summarized in isolated intense abdominal and/or thoracic pain. It also shows the absolute necessity, in traumatic pathology, to properly analyze the mechanism of injury to avoid an underestimation of the potential damage.
